# Access to health care in relation to socioeconomic status in the Amazonian area of Peru

**DOI:** 10.1186/1475-9276-8-11

**Published:** 2009-04-15

**Authors:** Charlotte Kristiansson, Eduardo Gotuzzo, Hugo Rodriguez, Alessandro Bartoloni, Marianne Strohmeyer, Göran Tomson, Per Hartvig

**Affiliations:** 1 IHCAR (Div International Health), Karolinska Institutet, Stockholm, Sweden; 2 Inst Med Trop A von Humboldt, Universidad Peruana Cayetano Heredia, Lima, Peru; 3 Health Directorate of Loreto, Iquitos, Loreto, Peru; 4 UFDID, University of Florence, Florence, Italy; 5 Dept Pharmacology and Pharmacotherapy, Farma, University of Copenhagen, Denmark

## Abstract

**Background:**

Access to affordable health care is limited in many low and middle income countries and health systems are often inequitable, providing less health services to the poor who need it most. The aim of this study was to investigate health seeking behavior and utilization of drugs in relation to household socioeconomic status for children in two small Amazonian urban communities of Peru; Yurimaguas, Department of Loreto and Moyobamba, Department of San Martin, Peru.

**Methods:**

Cross-sectional study design included household interviews. Caregivers of 780 children aged 6–72 months in Yurimaguas and 793 children of the same age in Moyobamba were included in the study. Caregivers were interviewed on health care seeking strategies (public/private sectors; formal/informal providers), and medication for their children in relation to reported symptoms and socio-economic status. Self-reported symptoms were classified into illnesses based on the IMCI algorithm (Integrated Management of Childhood Ilness). Wealth was used as a proxy indicator for the economic status. Wealth values were generated by Principal Component Analysis using household assets and characteristics.

**Results:**

Significantly more caregivers from the least poor stratum consulted health professionals for cough/cold (p < 0.05: OR = 4.30) than the poorest stratum. The poorest stratum used fewer antibiotics for cough/cold and for cough/cold + diarrhoea (16%, 38%, respectively) than the least poor stratum (31%, 52%, respectively). For pneumonia and/or dysentery, the poorest used significantly fewer antibiotics (16%) than the least poor (80%).

**Conclusion:**

The poorest seek less care from health professionals for non-severe illnesses as well as for severe illnesses; and treatment with antibiotics is lacking for illnesses where it would be indicated. Caregivers frequently paid for health services as well as antibiotics, even though all children in the study qualified for free health care and medicines. The implementation of the Seguro Integral de Salud health insurance must be improved.

## Background

Following health reforms in the 1980s and 1990s, many Latin-American countries moved from universal coverage (free health care financed by public funds) towards cost recovery initiatives utilizing, for example, user fees and social insurances [1-3]. However, user fees have been shown to represent an important barrier to accessing health services, especially for poor people[4]. Strategies including fee exemption – aimed at mitigating exclusionary effects – have proven to be stigmatizing and costly since administrational measures are needed to identify the poor [5,6]. It is likely, therefore, that if targeted interventions are to be effective in reaching the poor, special strategies need to be more carefully outlined [7,8].

In Peru, inequity in health service utilization during the late 1990s was shown for adults [9] but no corresponding figures for children have been presented. This lack of attention is surprising since death resulting from infectious disease remains a major health problem for children in low and middle income countries. In Peru, the most common causes of mortality among children under five years of age remain acute respiratory tract infections [10]. Furthermore, several studies have shown a well-established connection between socioeconomic status and health [11,12], where proximate factors, such as health prevention, nutrition and care, have been directly linked to socioeconomic status [13]. These studies make clear that in addition to ill health, the costs for health care and medicines further impoverish vulnerable population groups [14], poor children notwithstanding.

The Seguro Integral de Salud state health insurance (SIS) was implemented in Peru in 2001 and at the time of this study offered free of charge health care and pharmaceuticals, such as antibiotics, to children in the study area, regardless of socioeconomic status. Access to health services and pharmaceuticals following policy implementation has improved, at least theoretically through the SIS, for children from all socioeconomic groups; however, presumptive improvements in the health seeking behavior of poor children have yet to be empirically analyzed or verified. The question of verifiable improvements becomes even more essential when considering poor children who have high geographic access to health facilities. The aim of this study, therefore, was to describe health care access – measured as consultations with health professionals – and antibiotic use in relation to socioeconomic status for children who recently presented symptoms of infectious disease.

## Methods

### Study area

#### Population and socioeconomic structure

This survey was conducted in 2002 in two Peruvian communities, Yurimaguas (Department of Loreto) and Moyobamba (Department of San Martin). In 2002, each community had a population of approximately 32000 inhabitants. Both departments represent one of the most underprivileged areas of Peru – the Amazonian region. Approximately 55% of the population lives below the poverty line, and 15% in extreme poverty and the majority of the working population survive on subsistence farming [15]. Yurimaguas is less accessible than Moyobamba which has a better infrastructure and is easily reached from the surrounding communities.

#### Health services

The study was conducted in urban settings where geographical distances to health facilities are small. Yurimaguas has one Ministry of Health (MoH), a public hospital, a Local Committees for Health Administration (CLAS) Maternal Health Centre [16] and two health posts. In addition there is a Social Security Institute hospital (ES SALUD). In Moyobamba, there are three health posts and one health center. At the time of the study, the hospital in Moyobamba belonged to ES SALUD system, and the MoH facilities either paid the ES SALUD hospital for treatment of public sector patients or referred patients to the nearby city of Tarapoto. At both study locations, the hospital and health center employees mainly included medical doctors, while the health posts were staffed exclusively with nurses, midwives and health technicians.

All licensed, private pharmacies – nine in Yurimaguas and fifteen in Moyobambas – had trained pharmacists on their staff list. However, in most cases, these pharmacies were run by assistants without formal education. Officially, antibiotics could only be sold by pharmacies, but in practice they were also bought without prescription, available at the market place, in food stores or from traditional healers.

The SIS was created by merging the already existing Maternal/Infant and School insurances. In theory, SIS provided health care and essential pharmaceuticals via the public health care sector free of charge to those with low economic status, to pregnant women and to senior citizens, according to insurance schemes specified by target group. In areas with a poverty level higher than 60%, such as the Amazonian area, all children qualified for the insurance, irrespective of their parents' economic status. The insurance covered health care, including essential generic drugs and diagnostic services. Affiliation to the insurance was not, however, automatic and the children had to be registered every calendar year at the hospital's SIS office.

### Means of data collection

#### Design and sampling

The results presented in this paper were generated within the ANTRES project, a collaborative research project funded by the EC INCO-DEV, ICA4-CT-2001-1001, addressing the themes of antimicrobial use and resistance in Peru and Bolivia.

This cross sectional survey used household interviews. Faecal samples were also collected for microbiological studies, which have been reported elsewhere [17]. Children with three or more loose stools in the 24 hours prior to sampling (WHO definition of diarrhoea, 1993) were excluded from the study in order to ensure the implementation of the microbiological study within the same survey. A modified cluster sampling approach was used and included a total of 1600 children, aged 6 to 72 months. Eight hundred children from each village were sampled. Yurimaguas and Moyobamba were divided into zones of varying sizes, containing households distributed in blocks. A stratified sampling of blocks (within zones) was then conducted with a probability proportional to the size of the zone. Eighty clusters were sampled in each community, with each cluster consisting of 10 children (one per household) within a randomly assigned block. Within each block, the interviewers randomly chose a corner of the block and, pursuing a random direction from this corner, consecutively visited households until they had recruited ten eligible children. If they found no child aged 6 to 72 months, the interviewers proceeded to the next house, and likewise, if no eligible child was identified within a block, the interviewers proceeded to a nearby block, which had been mapped as a substitute. In total, 780 children from Yurimaguas and 793 children from Moyobamba were included in the study described in this paper.

### Interviews

Household interviews were conducted by ten trained interviewers from the public health sector (health technicians, nurses or midwives) who had extensive experience working with community outreach activities. The children's caregivers (mother, father, grandparent or other adult caregiver) were interviewed using a structured questionnaire (available from the first author) which had been pre-tested and validated during a pilot study. The caregivers were chosen for the interviews on the basis of being present in the household and taking care of the child during the time of the interviewers' visit. The majority of caregivers were mothers (87% in Moyobamba and 85% in Yurimaguas). Interview questions addressed the child's symptoms for the most recent illness during the previous two weeks, as well as all actions taken by the caregivers to cure the symptoms, including medication and healing practices. The interviewers first asked for the symptoms in an open-ended question and then probed by stating all symptoms in the questionnaire one by one while also explaining the symptoms.

A system for checking data quality was developed in order to ensure high quality during study implementation: on a daily basis, study supervisors screened the questionnaires for missing responses and logical inconsistencies and requested re-visits to households where more detailed clarifications where needed. The caregivers were also asked to show interviewers the package or blister pack if antibiotic use was reported. If caregivers were not in possession of the package or did not remember the name of the antibiotic consumed, but could describe the package, bottle or tablet, the interviewers presented them with identifiable antibiotics samples. The interviewers also carried a list of local antibiotic brand names and the corresponding ATC category [18].

The health seeking behavior was classified into "self-care", "exclusive consultation (with persons working with health issues)" and "self care and consultations". Self-care was defined in line with Levin [19] as all those activities undertaken to treat illness without professional assistance. We defined medical doctors, nurses and midwives, health technicians, pharmacy staff and traditional healers as professional assistance or, as stated in the questionnaire, "persons working with health issues".

### Illness classification

The symptoms (as reported by caregivers) were classified according to the principles of the IMCI algorithm (Integrated Management of Childhood Illness) for classification and treatment of infectious diseases in children in low malaria-prevalence areas [20]. The algorithm is based on the absence or presence of the following key symptoms: fast breathing, cough, diarrhoea, and/or blood in stool.

The children were classified with one of the following illnesses: diarrhoea (presence of: diarrhoea; absence of: blood in stool, cough and other symptoms of cold, 'fast breathing'), dysentery (presence of: blood in stool, diarrhea; absence of: cough and other symptoms of cold, 'fast breathing'), cough/cold (presence of: fever, cough and other symptoms of cold; absence of: diarrhoea, blood in stool, 'fast breathing'), pneumonia (presence of: 'fast breathing', cough; absence of: diarrhoea, blood in stool), cough/cold + diarrhoea (Symptoms of cough/cold and symptoms of diarrhea), pneumonia and/or dysentery (one of following three combinations: symptoms of pneumonia and diarrhoea, *or *symptoms of pneumonia and dysentery, *or *cough/cold and dysentery).

### Analysis of wealth status

In many low-income settings, it is problematic to use traditional measures, such as income or consumption, for the assessment of an individual's financial status, due to practical limitations of collecting accurate data [12]. In this study, wealth was used as a proxy indicator for the economic status, under the assumption that wealth is reflected in the assets owned within a household [21]. Information about household assets and characteristics was collected during the interviews. Principal component analysis (PCA) was used to generate scoring weights for each variable: the number of household members divided by the number of rooms in the house, the access to electricity, type of floor, type of toilet, type of water source, and the ownership of a fan, TV, motor-bike and refrigerator, using the first principal component. The scores were then summed up to assign a wealth index value to each household. The consistency of the PCA-assigned wealth with the ownership of assets was controlled by cross tabulations. No absolute cut-off points for the level of wealth could be assumed based on the wealth index values. Thus, the population was divided into quartiles based on the value of the wealth variable in order to capture relative variances. For the analysis presented in this paper, quartile number one (Q1) was considered as a proxy for the lowest wealth – the poorest, and quartile number four (Q4) as the least poor – the richest.

### Data analysis

The data from the questionnaires was introduced into an EPI INFO 2000 [22] database by double-entry and then compared. All discrepancies were verified with the paper originals and corrected. The database was exported to STATA, where it was scrutinised for quality and consistency prior to data analysis. Wealth variables were created separately for the data sets from Yurimaguas and Moyobamba by using the PCA. The respective wealth quarters from each community were then pooled to provide a data set large enough to allow for statistical analysis of variables related to health seeking behavior and use of medicines. The poorest quarter consisted of Q1 from Yurimaguas and Q1 from Moyobamba, and so forth.

The relation between wealth and health seeking behavior and antibiotic use was assessed. Logistic regressions were used to analyze differences between strata. Chi-square tests have been used to assess differences between the poorest half of the population (the two poorest strata pooled together) and the least poor half (the two least poor strata pooled) in relation to cost incurred for health care, cost for antibiotics and place of provision of antibiotics. The difference has been considered significant if the p-value is less than 0.05.

## Results

### Morbidity

Of a total of 780 children from Yurimaguas included in the study, 382 (48%) children had shown symptoms related to infectious illnesses in the previous two weeks. In Moyobamba, 425 (54%) of a total of 793 children had suffered from symptoms. In total, four children were excluded due to incomplete data regarding health seeking behavior, leaving 803 children for further analysis.

### Health seeking behavior

Many caregivers (42%) stated that they had consulted health professionals with regard to their children's health problems (Table 1). The least poor households had consulted health professionals for the non-severe illness cough/cold significantly more frequently than the poorest households (p < 0.05: OR 4.30). Similarly, for the non-severe illness, cough/cold + diarrhoea, least poor households had consulted health professionals to a greater extent (p < 0.05: OR 3.74). The least poor households also consulted more frequently with health professionals for severe illnesses, such as pneumonia and/or dysentery (OR = 2.33) and pneumonia (OR = 2.92), even though the difference was not significant.

**Table 1:** Children seeking care from health professionals in relation to self reported symptoms and wealth quarters

	**Children seeking care from health professionals**						
**Illness**	**Q1** % seeking care (total no children with illness)	**Q2** % seeking care (total no children with illness)	**OR**	**Q3** % seeking care (total no children with illness)	**OR**	**Q4** % seeking care (total no children with illness)	**OR**

**Cough/cold**	21% (99)	28% (116)	1.62	28% (128)	1.88*	51% (104)	4.30*
**Pneumonia**	33% (12)	82%(11)	5.25	50% (16)	1.94	71% (14)	2.92
**Cough/cold + diarrhoea**	35% (62)	46% (46)	1.70	41% (37)	1.62	50% (30)	3.74*
**Pneumonia and/or dysentery**	50% (18)	54% (13)	1.17	67% (9)	2	70% (10)	2.33
**Diarrhoea**	17% (18)	27% (22)	1.05	40% (10)	1.87	20% (15)	0.93

Total	209	208		200		173	

Self reported symptoms classified as illnesses based on principals from IMCI. Wealth quarters were defined by principal component analysis (PCA). Quartile 1 (Q1) represents the poorest quartile of the study sample (the relatively poorest caregivers), quartile 2 (Q2) the second poorest, quartile 3 (Q3) the second least poor quartile and quarter 4 (Q4) the least poor quartile. Amount of children seeking care from health professionals (pharmacy staff not included) is showed as percentage and total number in parenthesis. OR = Odds ratio. The Odds ratio is stated for each wealth quarter as compared to quarter 1. * = p < 0.05

In all strata, public sector medical doctors were the most commonly visited health professionals. The least poor households consulted nurses and health technicians to a lower extent (1% and 1%, respectively) than the poorest households (12% and 24%, respectively). Caregivers from all strata paid out-of-pocket for care provided by public sector health facilities (Table 2). In Yurimaguas, significantly more caregivers from the two least poor strata made out of pocket payments, as compared to the two poorest strata (p < 0.05). Also in Moyobamba more least poor caregivers made out of pocket payments, but the difference between strata was not significant.

**Table 2:** Cost incurred for public sector health care

	Cost incurred for public sector health care					
	**Yurimaguas**			**Moyobamba**		
	Free of charge	Paid out-of-pocket	Total	Free of charge	Paid out-of-pocket	Total

Poorest (Q1)	31 (86%)	5 (14%)	36	13 (87%)	2 (13%)	15
Q2	48 (92%)	4 (8%)	52	24 (92%)	2 (8%)	26
Q3	29 (74%)	10 (26%)	39	25 (81%)	6 (19%)	31
Least poor (Q4)	26 (67%)	13 (33%)	39	34 (81%)	8 (19%)	42

	134	32	166	128	30	158

Number of caregivers paying for the health care provided by the public sector health professionals stated in relation to wealth quartiles (as defined by principal component analysis (PCA)). Quartile 1 (Q1) represents the poorest quartile of the study sample (relatively poorest), quartile 2 (Q2) the second poorest, quartile 3 (Q3) the second least poor quartile and quarter 4 (Q4) the least poor quartile. Number of children whose caregivers paid for the health care provided by the public sector health professional or who were provided care free of charge, stated as numbers, with percentage of total number of children per quartile indicated in parentheses. Chi-square tests have been used to assess differences between two poorest (Q1 and Q2) and two least poor (Q3 and Q4) strata. Significant difference between strata was found for Yurimaguas (p < 0.05) but not for Moyobamba.

### Use of antibiotics

A similar antibiotic use was reported for the children in Yurimaguas and Moyobamba (42% and 36% respectively). As the patterns of medicine use in relation to socio-economic status and illnesses were similar for the two communities, the pooled data from both is presented in Table 3. For cough/cold, the least poor used significantly more antibiotics than the poorest (OR = 2.29). The same trend could be observed for pneumonia and/or dysentery, with the least poor using significantly more antibiotics (80%) than the poorest (16%).

**Table 3:** Antibiotic use in relation to self reported symptoms and wealth quarters

	**Use of antibiotics**						
**Illness**	**Q1** % using antibiotics (total no children with illness)	**Q2** % using antibiotics (total no children with illness)	**OR**	**Q3** % using antibiotics (total no children with illness)	**OR**	**Q4** % using antibiotics (total no children with illness)	**OR**

**Cough/cold**	16% (99)	30% (116)	1.85	39% (128)	2.17*	34% (104)	2.29*
**Pneumonia**	64% (12)	63%(11)	0.93	56% (16)	0.71	53% (14)	0.63
**Cough/cold + diarrhoea**	38% (62)	62% (46)	2.64*	47% (37)	1.41	52% (30)	1.73
**Pneumonia and/or dysentery**	16% (18)	80% (13)	21.33*	70% (9)	12.44*	80% (10)	21.33*
**Diarrhoea**	35% (18)	43% (22)	1.39	-(10)	-	39% (15)	0.93

Total	209	208		200		173	

Self reported symptoms were classified as illnesses based on principals from IMCI. Wealth quartiles were defined by principal component analysis (PCA). Quartile 1 (Q1) represents the poorest quartile of the study sample (the relatively poorest of the caregivers), quartile 2 (Q2) the second poorest, quartile 3 (Q3) the second least poor quartile and quarter 4 (Q4) the least poor quartile. Amount of children treated with antibiotics, including through self medication, showed in percentage, total number of children shown in parentheses. OR = Odds ratio showing each wealth quarter as compared to quarter 1. * = p < 0.05

### Location of antibiotic acquisition

The locations for antibiotic provision, including antibiotics for self-medication, were investigated. In Yurimaguas, the two least poor strata had bought their antibiotics (either as a part of self care or with a prescription) at the pharmacy (private or public pharmacy) significantly (p < 0.05) more frequently (45%) than the two poorest quartiles (15%) (Table 4). The least poor group received their antibiotics free of charge by the health insurance system (40%) less frequently than the poorest quartile (65%). In Moyobamba, the least poor caregivers more frequently (58%) received antibiotics free of charge from the insurances than the poorest group (46%).

**Table 4:** Place of acquisition of antibiotics in relation to wealth quarters

	**Place of antibiotic acquisition in Yurimaguas**						**Place of antibiotic acquisition in Moyobamba**					
	Public pharmacy	Private pharmacy	Market place	Free of charge through insurance	Other	Total	Public pharmacy	Private pharmacy	Market place	Free of charge through insurance	Other	Total

**Poorest (Q1)**	2(6%)	3 (9%)	5 (15%)	22 (65%)	-	32	8 (29%)	5 (18%)	1 (4%)	13 (46%)	1 (4%)	28
**Q2**	5 (10%)	7 (14%)	3 (6%)	33 (66%)	1 (2%)	49	3 (7%)	15 (38%)	4 (10%)	17(43%)	1 (3%)	40
**Q3**	7 (19%)	11 (31%)	1 (3%)	15(42%)	1 (3%)	35	7 (17%)	12 (29%)	2 (5%)	19 (45%)	2 (4%)	42
**Least poor (Q4)**	9 (24%)	8 (21%)	1 (3%)	19 (40%)	1 (3%)	38	10 (25%)	5 (13%)	-	23 (58%)	2 (6%)	40

Total	23	29	10	89	3	154	28	37	7	72	6	150

Wealth quarters were defined by principal component analysis (PCA). Quartile 1 (Q1) represents the poorest quartile of the study sample (the relatively poorest caregivers), quartile 2 (Q2) the second poorest, quartile 3 (Q3) the second least poor quartile and quarter 4 (Q4) the least poor quartile. Number of children stated per place of provision, percent of total number of children per quartile and community shown in parentheses. Chi-square tests have been used to assess differences between two poorest (Q1 and Q2) and two least poor (Q3 and Q4) strata. Significant difference between strata for antibiotic acquisition from pharmacies was found for Yurimaguas (p < 0.05) but not for Moyobamba.

The cost associated with antibiotics prescribed by public sector health professionals was investigated. In Yurimaguas, the two least poor strata had made out-of-pocket payment for the antibiotics to a significantly larger extent (p < 0.05) than the two poorest strata (Table 5). In Moyobamba, there was no significant difference between strata.

**Table 5:** Type of financing for antibiotics prescribed by public sector health professionals, in relation to wealth quarters

	Financing for antibiotics prescribed by public sector					
	**Yurimaguas**			**Moyobamba**		
	Free of charge	Paid Out-of-pocket	Total	Free of charge	Paid Out-of-pocket	Total

Poorest (Q1)	21 (91%)	2 (9%)	23	12 (86%)	2 (14%)	14
Q2	33 (92%)	3 (8%)	36	18 (100%)	-	18
Q3	15 (75%)	5 (25%)	20	15 (71%)	6 (29%)	21
Least poor (Q4)	15 (54%)	13 (46%)	28	18 (72%)	7 (28%)	25

	84	23	107	63	15	78

Wealth quarters were defined by principal component analysis (PCA). Quartile 1 (Q1) represents the poorest quartile of the study sample (the relatively poorest caregivers), quartile 2 (Q2) the second poorest, quartile 3 (Q3) the second lest poor quartile and quarter 4 (Q4) the least poor quartile. Number of children receiving antibiotics prescribed by public sector health professionals provided free-of-charge or in return for out-of-pocket payment stated as numbers, with percentage per quartile and community shown in parentheses. Chi-square tests have been used to assess differences between two poorest (Q1 and Q2) and two least poor (Q3 and Q4) strata. Significant difference between strata was found for Yurimaguas (p < 0.05) but not for Moyobamba.

## Discussion

This study shows that the poorest households consulted health professionals for their sick children less frequently. The poorest children were provided with fewer antibiotics for illnesses where the IMCI algorithm recommended antibiotic use. Inequity in access to health care, here measured as consultations with health professionals, prevailed in an urban setting despite of high geographical access to health facilities and the SIS health insurance providing free health care to children. The high theoretical access to health care makes this study unique from other studies focusing on Latin American countries where similar inequitable results have been shown [9,23].

During the study period, the recently introduced Peruvian state health insurance Seguro Integral de Salud (SIS) aimed to reduce financial barriers to health by providing free health care and pharmaceuticals for the target groups. In the Amazonian area, all children qualified for the SIS due to the high poverty status of the region. However, the formal supply of free health care was not enough to ensure equitable access, and instead, remaining barriers exist which prevented equitable supply. For example, following initiation of the SIS, it is plausible that caregivers lacked adequate information about the benefits and regulations of the new SIS insurance system or that they may have confused it with previous insurance schemes that were granted only after assessment of the families' financial status. Either way, during the implementation of this study, the local health professionals complained that the poorest households were "over-seeking" health care for unnecessary symptoms and thereby wasting resources. Ironically, this attitude would have contributed to preventing truly vulnerable patient groups from seeking health care. Moreover, caregivers' perceptions about quality of care and pharmaceuticals, as well as attitudes of health professionals, have been shown to be important factors determining health facility utilization [24-27].

The analysis of costs associated with health care services provided by the public health facilities in the two study sites showed that payments were made for consultations with health professionals, more often in Yurimaguas than in Moyobamba. Cost has been shown to be an important factor influencing health seeking behavior [28]. According to information from key informants, as well as the first author's own observations, health facilities in several regions of Peru excluded, based on cost, some aspects of treatment from the SIS benefits, such as diagnostic services, or limited the amount of beneficiaries per day. Reimbursement procedures were also too unclear and facilities feared losing money on the SIS patients. Instead, the patients had to pay services out-of-pocket, which could have caused the poorest group to avoid seeking care rather than facing the risk of unexpected costs. The difference in cost charges between Yurimaguas and Moyobamba could have been the result of the respective communities' interpretation and implementation of the SIS insurance and its benefits at the health facility level. Another important barrier at this level is the time consuming nature of administrational procedures as related to SIS affiliation. It is further possible that people chose to pay for care rather than losing valuable hours waiting in line at the SIS office. The influence of similar health systems constrains on effective service delivery and health seeking behavior has been underlined by other researchers [29].

The poorest children were provided with fewer antibiotics for some of the illnesses where antibiotic use was recommended in the IMCI algorithm. According to our definition, this disadvantage represents a link to the inequity in heath-seeking behavior. An analysis of the costs paid for the antibiotics and the health care provided by public sector health professionals showed that a number of caregivers had paid for their antibiotics, even though these were supposed to be provided free of charge to all children through the SIS insurance. Our assessment of the location where the antibiotics had been acquired showed that a large part of the antibiotics were bought at public pharmacies. This finding indicates that the SIS implementation was not functioning in an optimal manner, as the health facilities were still charging the patients. On the other hand, purchasing antibiotics rather than receiving them free of charge could also be an indication of problems related to out-of-stock pharmaceuticals in the public sector or that informal payments were charged.

The poorest were mainly self caring for non-severe illnesses such as common cold or non-complicated diarrhea. This is in line with IMCI recommendations [20] and can be considered as rational. In contrast, the least poor frequently sought health care for the same illnesses where consultations were not really necessary. By doing this, they also contributed to a waste of financial resources allocated to health care services. Of note, the least poor children were also frequently given antibiotics for illnesses where it was not indicated according to the IMCI guidelines. This trend coincided to their frequent health care consultations, as the majority of antibiotics were prescribed by the health professionals.

## Conclusion

This study shows that even in a setting providing universal access to free health care for children, the poorest seek less care from health professionals for severe illnesses, and that treatment with antibiotics is lacking for illnesses where it should otherwise be indicated. Despite that all children in the study qualified for free health care and medicines, their caregivers frequently paid for health services as well as antibiotics. Given these findings, the implementation of the Seguro Integral de Salud health insurance must be improved.

## Competing interests

The authors declare that they have no competing interests.

## Authors' contributions

CK participated in the design and implementation of the study, acquisition of data, analysis and interpretation of data and helped to draft the manuscript. EG participated in the design and coordination of the study, analysis and interpretation of data and helped to draft the manuscript. HR, AB and MS participated in the design and implementation of the study, acquisition of data and helped to draft the manuscript. GT and PH participated in the analysis and interpretation of data and helped to draft the manuscript. All authors read and approved the final manuscript.
